# Detection of Tomato Ringspot Virus Based on Microfluidic Impedance Sensor

**DOI:** 10.3390/mi13101764

**Published:** 2022-10-18

**Authors:** Chen Li, Bo Ye, Yongxin Xi, Mu Yuan

**Affiliations:** 1 College of Quality and Safety Engineering, China Jiliang University, Hangzhou 310018, China; 2 Zhejiang Hechuan Technology Co., Ltd., Quzhou Haichuang Park, Wenyi Road, Hangzhou 324400, China

**Keywords:** microfluidic chip, impedance sensor, tomato ringspot virus, quantitative detection

## Abstract

A microfluidic impedance sensor embedded with gold interdigitated array microelectrodes was utilized to rapidly detect Tomato Ringspot Virus (ToRSV) and achieve efficient and precise detection. The electrochemical impedance spectrum was obtained by immobilizing ToRSV antibody on the surface of a gold interdigital array microelectrode and mixing it with ToRSV to generate an impedance change. The electrochemical impedance spectrum was obtained. The equivalent circuit was established to analyze the mechanism of impedance change, and the quantitative linear relationship between ToRSV concentration and impedance was established. According to an equivalent circuit analysis, ToRSV increases the solution resistance Rs, the electron transfer resistance Ret on the electrode surface, and the double layer capacitance Cdl, resulting in an increase in impedance. The results reveal that the ToRSV concentration detected in the range of 0.001 to 10 μg/mL ranges from 248.8 to 687.2 kΩ at the ideal detection frequency of 10.7 Hz, with a good linear connection, R^2^ = 0.98. When this method’s detection limit is tested, the impedance value is 367.68 kΩ. 0.0032 μg/mL was the detection limit. The sensor is quick and easy to use, has high detection sensitivity, and can be used to detect other plant viruses.

## 1. Introduction

In recent years, as China’s foreign trade continues to expand, international travel becomes more convenient, and cross-border e-commerce continues to develop, the biosecurity situation on China’s customs coast is becoming increasingly critical. There has been an unprecedented increase in the number and types of foreign harmful organisms, endangering our country’s agriculture and ecological environment, and even undermining economic and social stability and threatening national security. Tomato ringspot virus (ToRSV), as an important crop pathogen, can infect more than 150 crops, such as soybeans, tomatoes, tobacco, grapes, and apples, and cause serious diseases [1,2]. At present, ToRSV has become a class I quarantine virus that is prohibited from entering China [3].

The common method for testing ToRSV at home and abroad is polymerase chain reaction (PCR). Robert R Martin et al. [4] used collagenase to improve detection of ToRSV virus in vector nematodes by RT-PCR. Elwin L S et al. [5] used real-time quantitative RT-PCR SYBR Green assay to develop the grape ToRSV virus. Qian-Qian Yang et al. [6] developed a fast reverse transcription-cross-priming amplification (RT-CPA) coupled with lateral flow dipstick (LFD) diagnostic method for BPMV detection. The RT-CPA-LFD assay targeting the BPMV coat protein gene is highly specific for the diagnosis of the ToRSV virus transmitted from soybean seeds. The sensitivities of the real-time fluorescent RT-CPA and the RT-CPA-LFD assay were at least 50 pg/μL and 500 pg/μL, respectively. W G Wang [7] used semi-nested RT-PCR method to detect tobacco ringspot virus. The results showed that the detection sensitivity of DA-SELISA was similar to that of RT-PCR, while the detection limit of semi-nested RT-PCR was 10 ng/mL, which was 10^3^ times higher than that of the two methods. PCR-based methods can detect tomato ringspot virus quickly and accurately with specificity and low detection limits. However, when the PCR method is used to detect ToRSV, the instrumentation cost of the method is relatively high considering the large number of samples that need to be tested by customs, and in addition, PCR is sometimes prone to false positive results due to DNA contamination [8].

Therefore, there is a need to establish a rapid, simple and sensitive field detection method for ToRSV. The microfluidic technology that has received much attention in recent years can make up for this shortcoming. The greatest advantage of microfluidic chips is the creation of a controlled microenvironment that precisely drives and controls microfluidic flow in microchannels, resulting in increased detection sensitivity. In addition, all analytical processes, including sample preparation, reaction, separation and detection are integrated into a single microfluidic chip, facilitating instant detection in the field [9,10,11]. Meanwhile, microfluidic impedance sensors consisting of this technology combined with electrochemical impedance detection methods, impedance-based biosensors [12], as a type of electrochemical biosensors, have shown their good advantages in terms of speed, accuracy and sensitivity in the detection of pathogens in the field [13]. A number of emerging microfluidic chips have been successfully developed for the detecting pathogens. For example, Wang C H et al. [14] designed a nanomaterial-based micro-finger structure for the detection of Staphylococcus aureus and analyzed the results to obtain a detection limit of 43 CFU/mL for the sensor. Zheng L et al. [15] developed a novel biosensor using gold nanoparticles (AuNPs) for the indication of different concentrations of *E. coli* O157:H7. The sensor showed good specificity and sensitivity for *E. coli* O157:H7 in chicken samples with a detection limit of 50 CFU/mL. Jian F et al. [16] detected Salmonella using screen-printed carbon electrodes coated with gold nanoparticles (rGO/AuNPs) with a detection limit of 89 CFU/mL.

In conclusion, microfluidic impedance sensors in the field of biological field detection are a research hotspot in recent years, the sensor has rapidity, specificity, low consumption of experimental samples, low construction cost, simple assembly it leads to easy miniaturization and development of automated instruments, is the ideal detection method to achieve rapid detection of biological viruses in the field [17,18,19]. In this paper, a microfluidic chip with a gold interdigital array microelectrode was designed and produced in this research to address the ToRSV detection challenge, and a microfluidic impedance sensor detection platform for ToRSV detection was built. The equivalent circuit is used to describe the mechanism of impedance formation and change, as well as perform specialized detection tests, and the impedance spectrum is acquired using the electrochemical detection principle. Finally, a quantitative model of the relationship between ToRSV concentration and impedance is made. This gives ToRSV detection in the field a theoretical and technical basis.

## 2. Materials and Methods

### 2.1. Principle of Microfluidic Impedance Detection

The microfluidic impedance detection technology is based on a microfluidic chip device that turns biological viral concentration into an electrochemical impedance signal for fast detection. The detection principle is based on the microfluidic chip system. The biological virus antibody molecule is placed on the surface of the conductive transducer (golden interdigital array microelectrode) in the microfluidic chip, and then the antigenic solution of the biological virus is injected into the mesoscopic microchannel. In the microchannel, the viral antigen antibody binds to the conductive transducer, forming a particular complex that alters the conductive transducer’s impedance characteristics. To achieve the detection goal, a quantitative link between the change in impedance signal and the concentration of the detection chemical is created [20].

Electrochemical impedance spectroscopy (EIS) was used for the impedance measurements performed in this paper. Electrochemical impedance spectroscopy, earlier known as AC impedance, was originally used in electricity to study the response characteristics of linear circuit networks at frequencies, and was later applied to electrochemical research by the Dutch physical chemist Sluyters, and has now become an important method for electrochemical studies of electrode processes. The impedance spectrum is obtained by applying a small amplitude sinusoidal alternating excitation voltage perturbation signal to the study system and measuring the variation of the ratio of AC voltage to current signal with the frequency (or phase angle) of the sinusoidal wave.

### 2.2. Preparation of Tomato Ringspot Virus

#### 2.2.1. Materials

The test Tomato Ringspot Virus (ToRSV), Tobacco Ringspot Virus (TRSV), Southern Bean Mosaic Virus (SBMV), and Arabis Mosaic Virus (ArMV) samples were provided by the Beijing customs port. The virus samples were lyophilized leaves of the respective hosts infected by each virus, inactivated by heat treatment, placed in sealed containers, dried with anhydrous calcium chloride, and stored at low temperature for transportation.

#### 2.2.2. Method

(1) We added 500 μL Plant Protein Extract A, 4 μL Protein Stabilizer, and 2 μL Protease Inhibitor (the above chemicals are from Bioengineering (Shanghai) Co., Ltd.’s plant protein extraction kit C500053) to a 1.5 mL centrifuge tube and mixed well with a constant temperature mixer (Model MSC-100, Hangzhou Ausheng Instrument Co., Ltd. (Block 9, No. 7, Turnang Science and Technology Economic Block, West Lake District, Hangzhou, Zhejiang Province, China));
(2) Liquid nitrogen was poured into the mortar, the tomato ringspot virus-infected plant leaves were added, and everything was thoroughly crushed into the previously prepared 1.5 mL centrifuge tube;
(3) The centrifuge tube was placed in a 4 °C mixed instrument at an oscillation speed of 800 rpm for one hour. The tube was then centrifuged for 15 min at 4 °C at a speed of 12,000 revolutions per minute in a frozen centrifuge (Model 5418R, Eppendorf Company, Hamburg, Germany);
(4) In the last step, the supernatant was transferred from the centrifuge tube into a fresh 1.5 mL centrifuge tube, 1 μL of the separated supernatant was taken, and an ultra-micro spectrophotometer was used to measure the tomato ringspot virus concentration, which was 26.6 mg/mL (model Nanodrop 2000, Thermo Fisher Scientific, Waltham, MA, USA). Before being kept at −80 °C, the PBS was diluted to 10, 1, 0.1, 0.01, and 0.001 μg/mL.

### 2.3. Microfluidic Impedance Detection Chips and Systems

**Figure 1:**
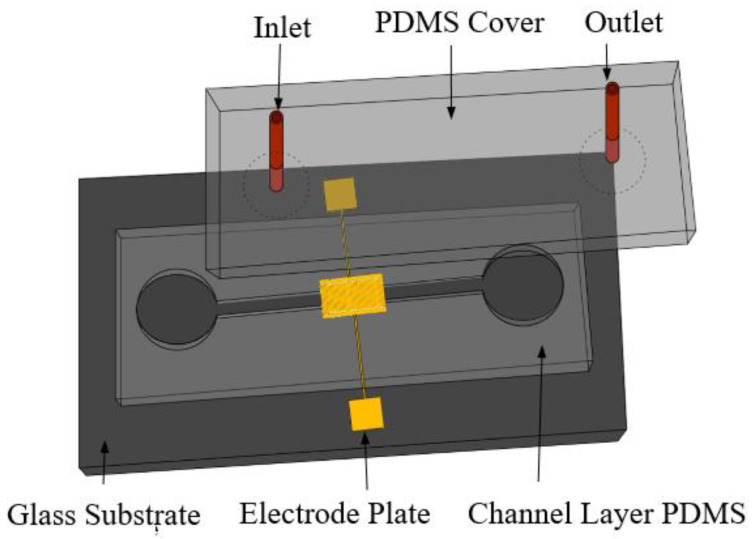
Schematic of microfluidic chip.

The microfluidic impedance detection chip designed in this paper was divided into three layers: the substrate layer, the channel layer, and the cover layer, as shown in Figure 1.

An improved gold foil and glass bonding efficiency was achieved by magnetron RF sputtering a 10 nm-thick Chromium (Cr) layer onto the surface of the glass substrate [21]. On top of the Chromium (Cr) layer, a gold (Au) thin film with a thickness of 100 nm was sputtered, and the electrode design for the micro-fork finger array was etched using a wet etching method. The channel layer comprised PDMS material, which was carved out of the micro-passage of the SU-8 positive film. Microchannels, a liquid storage pool, and a waste liquid pool made up the channel layer. In the same space, the microchannel and gold (Au) interdigital electrode formed a micro-reaction chamber. The antibody–antigen immune binding and electrode detection impedance signals were carried out in this region. The reaction space was sealed by the cover layer, which comprised PDMS material and covered by the channel layer. When the PDMS channel layer and glass substrate were treated with oxygen plasma, the surface hydroxyl groups increased, which greatly promoted the surface hydrophilicity. Due to the action of active silicon hydroxyl, the surface of PDMS channel layer and glass substrate underwent a condensation reaction, so as to realize the permanent bonding between the channel layer and the substrate. Similarly, the same was true for the cover layer and the channel layer. The PDMS cover with inlet and outlet connectors was then bonded to the first PDMS cover using oxygen plasma. The fluidic connectors were further sealed using epoxy glue in order to improve the device reliability and eliminate any possible fluid leakage.

**Figure 2:**
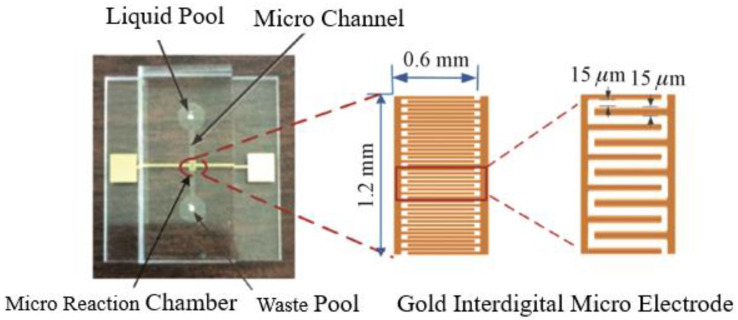
Schematic of gold interdigital microelectrodes.

The design of the gold (Au) interdigitated microelectrodes is shown in Figure 2. The following are the design parameters: 20 pairs of gold (Au) finger microelectrodes with an electrode thickness of 100 nm, a length of 0.6 mm, a width of 15 μm, and an electrode spacing of 15 μm. The microchannel’s dimensions were 7 mm in length, 0.5 mm in width, and 100 μm in depth; the parameters of the liquid storage pool and waste liquid pool were as follows: diameter of 3 mm and a depth of 100 μm; the volume of the microreactor was 1.2 mm × 0.5 mm × 0.1 mm = 0.06 mL. A 0.5 mm diameter hole was punched in the center of the cover layer corresponding to the liquid storage pool and the waste liquid pool. The connecting pipe was used for the input and output of the reagent.

**Figure 3:**
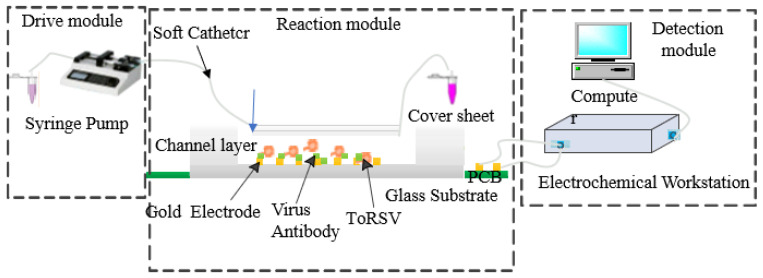
Schematic diagram of the microfluidic impedance detection system.

Figure 3 depicts the schematic diagram of the microfluidic chip impedance detection system, which consists primarily of the driving, reaction, and detection modules. The drive module for the microinjection pump (Model Harvard, Harvard Apparatus, Holliston, MA, USA). The injection pump connects to the entrance of the microfluidic chip through a soft catheter and a steel needle of 0.5 mm in diameter, in order to inject the virus detection sample and related reagents into the microfluidic chip at the prescribed rate. The reaction module consists of a microfluidic chip and a gold interdigital electrode that serve as a reaction platform for the development of phenomena in the sample capture and immune reaction processes, as well as the generation of impedance change signals. An electrochemical workstation (Model CS350, Wuhan Coster Technology Co., Ltd., Wuhan, China) and a computer make up the detection module. The gold fork micro-electrode of the microfluidic chip is linked to the electrochemical workstation, and the impedance change signal created on the gold fork micro electrode is detected, and the data are evaluated by the computer’s accompanying software.

### 2.4. Experimental Methods

The injection flow rate of the syringe pump was set to 0.00001 L/min to promote uniform distribution of ToRSV in the microfluidic chip and to ensure a continuous and specific reaction between ToRSV and the antibody. The electrochemical workstation impedance test parameters were set to: the alternating current (AC) excitation voltage is 100 mV and the test frequency is 1~100 kHz. Experiment with the following steps:

1. Measurement of the bare electrode: The impedance of the bare electrode was measured by injecting a PBS buffer. After the measurement was over, the microchannel and microelectrode were cleaned for 3 min with ultra-pure water;
2. Antibody immobilization: the micro channel was filled with 15 μL of ToRSV monoclonal antibody (0.5 mg/mL) and set up for 2 h. The pH value of the PBS buffer solution at this moment was 7.4 and the ion concentration was 0.1 mol/L. Immobilization of antibodies by natural sedimentation adsorption, a physical adsorption technique that is relatively easy to use, had minimal impact on the activity of biomolecules compared to other techniques, and reduced the likelihood of non-specific immunity occurring. The unabsorbed antibody was rinsed with ultrapure water after 2 h, and the impedance was measured by injection of PBS buffer. The cleaning procedure was repeated after the measurement;
3. (Bovine serum albumin)BSA sealing: 15 μL 1% BSA solution was injected and let to sit for 30 min. This shut the surface of the gold electrode that was not in contact with the antibody, preventing interference signals. BSA was washed away after 30 min, and PBS buffer was injected to determine the impedance value. Cleaning activities were repeated after the measurement was finished;
4. Detection of ToRSV: 15 μL of ToRSV sample was injected and let to sit for 30 min, then the impedance was tested. The cleaning operation after each measurement was repeated until the impedance was similar to that of a bare electrode.

The microfluidic chip designed in this paper was used for all experiments at one time; considering the demand of Beijing Customs for the detection of ToRSV in different samples to eliminate the possibility of cross-infection of different samples, the biosensor was selected as a disposable consumable, and the reproducibility of the chip was not considered in this paper.

## 3. Results and Discussion

### 3.1. Impedance and Phase Angle Analysis

**Figure 4:**
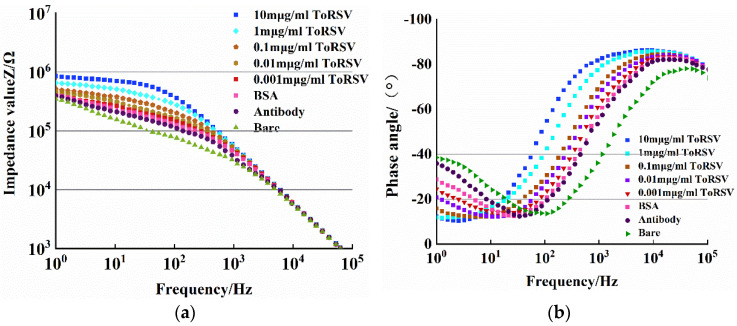
Impedance–frequency and phase angle–frequency curves of different solutions. (**a**) Impedance–frequency. (**b**) Angle–frequency.

Using the CS350 electrochemical workstation, the average impedance and phase values of 50 points were measured for three different batches of microfluidic chips, and the following electrochemical impedance Bode plots were generated to obtain the impedance versus detection frequency and phase angle versus detection frequency curves, as shown in Figure 4.

Figure 4a shows the impedance values of the bare electrode, antibody fixation, BSA closed electrode, and different concentrations of virus were evaluated in the range of 1~10^5^ kHz. Because of the influence of the detecting frequency on the impedance, the impedance–frequency trend is as follows: In the high frequency range of 1~100 kHz, the impedance value increases linearly with the decrease in the frequency, and the magnitude of the impedance value recorded at each step is not obviously different; in the low frequency range of 1~1000 Hz, the impedance tends to be stable with the frequency decreasing. In addition, compared with the bare electrode, the electrode impedance was further increased after single PBS buffer solution, antibody fixation, and BSA sealed electrode, demonstrating that the antibody was successfully fixed on the electrode surface. The electrode impedance value grows as the antibody collects ToRSV and reacts, and the higher the ToRSV concentration, the greater the increase in resistance. The reason for the increase in impedance value is that the antibody on the electrode surface binds to the virus antigen, which leads to the obstruction of ion flow and electron transfer between the electrodes, and the virus concentration is proportional to the obstruction ability. The higher the virus concentration, the greater the degree of obstruction to ion flow and electron transfer ability, resulting in increased electrode impedance.

**Figure 5:**
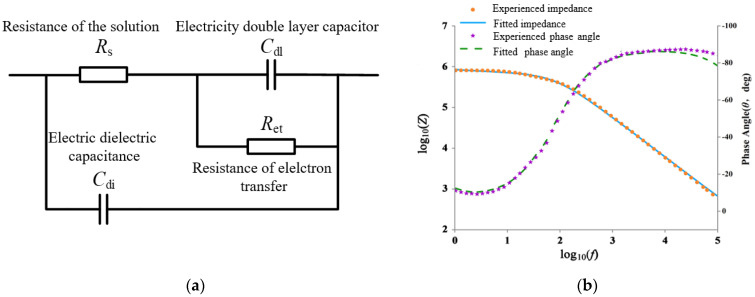
Equivalent circuit and simulation result. (**a**) Diagram of equivalent circuit. (**b**) Fitted curves of impedance and phase angle of 10 μg·mL^−1^ ToRSV.

Figure 4b shows that when the detection frequency range is 1~10^5^ Hz, the phase angle curves of different virus concentrations at different stages, such as bare electrode, antibody fixation, and BSA sealing electrode, when the phase angle is −90°, the system is a pure capacitance system, and when the phase angle is 0°, the system is a pure resistance system [22]. According to Figure 5, the maximum phase angle in the high frequency range (1~100 kHz) is close to −90°, while the minimum phase angle in the lower frequency range (1~10^3^ Hz) is −10~−40°, indicating that the generation of impedance is dominated by capacitance (capacitance characteristic) in the high frequency range, while in the low frequency region the impedance (resistance characteristic) dominates.

### 3.2. Equivalent Circuit Analysis

In order to better analyze the electrochemical impedance spectra in high-frequency and low-frequency areas, the equivalent circuit model of ToRSV detection was established. The equivalent circuit model, shown in Figure 5a, includes solution resistance (R_s_), electron transfer resistance (R_et_), electric double layer capacitance (C_dl_), and dielectric capacitance (C_di_). When the electrode is immersed in the electrolyte, the electrode surface and the surface of the solution are like a capacitor plate, and two charges are generated on the surface of the electrode at the same time, thus forming a double layer capacitance ± C_dl_; the double layer capacitance C_dl_ represents the effect of ions on the capacitance at the interface between the electrode and the solution; the electron transfer resistance (R_et_) represents the resistance of electron transfer to the solution on the surface of the Ret electrode; and characterization of the impedance formed at the interface between the solution and the gold-interdigitated electrode surface. R_s_ represents the resistance of the test solution, the resistance (R_s_) of the bacterial suspension is influenced by the bacterial surface charge and the ionic metabolites released by the bacteria in their surrounding liquid environment, which is connected in series with a parallel circuit of a double-layer capacitor C_dl_ and an electron transfer resistor Ret. C_di_ characterizes the dielectric capacitance of the test solution in parallel with the circuits of the solution resistor R_s_, the double-layer capacitor C_dl_, and the electron transfer resistor R_et_. The ZVIEW electrochemical impedance analysis software is used to establish an equivalent circuit model, and the complex nonlinear least square method is used to fit the equivalent circuit of 50 data points. The fitting is performed by Levenberg–Marquardt iterative fitting, and the fitting curves obtained are formulas (1) and (2). In the comparison of the fitting value and the measured value, the fitting curve of different concentrations is highly consistent with the measured value. Therefore, the impedance value and phase angle measured data of 10 μg/mL viral antigen are selected to analyze the effectiveness of the equivalent circuit fitting curve, as shown in Figure 5b. By comparing the fitting curve with the measured curve, it can be obtained that the average value of the absolute error of the impedance value is 0.8%, and the maximum error is 11.9%, the mean value of absolute error of the phase angle is 0.7°, and the maximum error is 5.5°. The measured and fitting tests are carried out on viral antigens with concentrations of 1, 0.1, 0.01, and 0.001 μg/mL, respectively. The average absolute values of impedance error are 0.6%, 1.1%, 1.2%, and 0.5%, respectively. The average absolute values of phase angle fitting error are 0.8°, 0.8°, 0.5°, 0.8°, and 1.4°, respectively. The experimental results show that the fitting curve has a good fitting effect, and the equivalent circuit model designed in this article has good fitting consistency with the microfluidic impedance sensor.

Two branches in the equivalent circuit, R_s_ + R_et_ + C_dl_ and C_di_, the control current flows in two different frequency range. In the low frequency region (1~1 kHz), the applied voltage frequency is not enough to make the current pass through the lipid membrane of the virus shell, so the captured virus is equivalent to the insulators. The equivalent circuit is equivalent to the current without the dielectric capacitance C_di_. The dielectric capacitor C_di_ does not perform work, the production of impedance is dominated by the R_S_ + R_et_ + C_dl_ branch, and its impedance value calculates the formula (2). In the high frequency region (1~100 kHz), the applied voltage frequency is sufficient to cause the current to pass through the lipid film of the viral housing, so that the current flows through the C_di_ branch. At this time, the R_S_ + R_et_ + C_dl_ branch does not work. The impedance generation of the C_di_ branch dominates, and its impedance value calculates the formula (3).

The parallel element impedance value of the electronic transfer resistor and the two-layer capacitor R_et_//C_dl_ calculates the resistance and capacitance and the formula of the impedance calculation formula.

(1) $$Z=R_{\mathrm{et}}/\sqrt{{(1+{2\pi \mathrm{fR}}_{\mathrm{et}}C_{\mathrm{dl}})}^{2}}$$

In the formula, Z represents the impedance value, ω; f represents frequency, Hz; R_et_ is electronic conversion resistance, Ω; C_dl_ is a dual-electrocomputer capacitor, NF.

At low frequencies, $2{\pi \mathrm{fR}}_{\mathrm{et}}C_{\mathrm{dl}}$ is much smaller than 1, and the parallel element impedance value Z of R_et_//C_dl_ can be equal to R_et_, so the impedance value generated by the series circuit of the solution resistance R_S_ and Ret//Cdl is calculated to be equal to two resistive element impedance values. Equation (2); calculated formula, such as (3)

(2) $$Z_{1}=R_{s}+R_{\mathrm{et}}{//C}_{\mathrm{dl}}=R_{s}+R_{\mathrm{et}}/\sqrt{{(1+{2\pi \mathrm{fR}}_{\mathrm{et}}C_{\mathrm{dl}})}^{2}}$$

(3) $$Z_{2}={1/(2\pi \mathrm{fC}}_{\mathrm{di}})$$

In the formula, Z_1_ represents the impedance generated by the R_s_ + C_dl_ + R_et_ branch circuit in the low-frequency zone, Ω; Z_2_ represents the impedance generated by the C_dl_ branch in the high-frequency zone, Ω.

### 3.3. Detection of ToRSV Concentration

The detection concentration of ToRSV is determined by the impedance response relationship at a certain detection frequency. Selecting the appropriate detection frequency can obtain a better impedance response, thereby optimizing the sensor. In the adjacent frequency gradient, the greater the impedance difference, the better the detection frequency.

**Figure 6:**
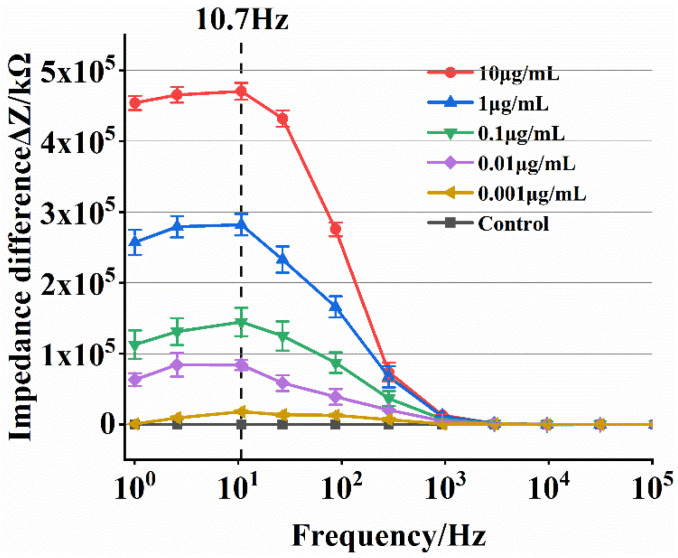
Impedance difference graph of different concentrations of virus.

The mean impedance difference curve for various ToRSV concentrations is shown in Figure 6. The mean impedance difference value can be calculated by subtracting the impedance values measured by the negative control group from the impedance values of three repeated ToRSV tests, and the standard error of the mean impedance difference is described by the error bars. The change in average impedance difference has the same trend: In the low frequency region (1~1 kHz), the average impedance difference increases first and then decreases. The maximum average impedance difference is at 10.7 Hz frequency, and the average impedance difference between adjacent concentrations is the largest at this frequency. In the high frequency region (1~100 kHz), the average impedance difference almost coincides with 0. This demonstrates that the detection frequency of 10.7 Hz is optimal for the microfluidic impedance sensor developed in this research.

**Figure 7:**
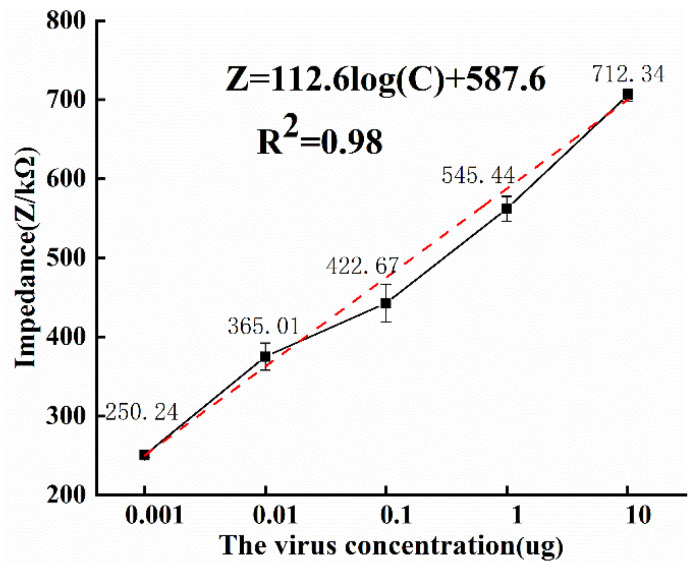
Diagram of concentration–impedance at the optimal detection frequency.

At an optimal detection frequency of 10.7 Hz, Figure 7 depicts the relationship between the impedance response values for each of the five virus concentrations evaluated. Considering that the biosensor is a disposable consumable, the experiment was repeated three times for the same virus concentration using different batches of microfluidic chips. The average impedance values of the three measurements were taken separately to obtain the average impedance values of 250.24, 365.01, 422.67, 545.44, and 712.34 kΩ for the five concentrations, and the error bars are shown as the standard errors describing the average impedance values.

The black lines in Figure 7 indicate the relationship curves of the average impedance response values for five different concentrations of ToRSV, and it can be obtained that the magnitude of the average impedance value increases with the increase in ToRSV concentration. The red dashed line in Figure 7 indicates the linear regression equation after linear fitting, and the correlation coefficient of the linear equation is 0.98, which shows a good linear law. Taking the average impedance value as the experimental value Z, the virus concentration C and the impedance value Z can be expressed as.

(4) $$Z=112.6\log(C)+587.6\;(R^{2}=0.98)\;$$

(5) $$\mathrm{LOD}=\frac{\;3\sigma }{S}$$

The limit of detection (LOD) [23,24] can be calculated from Equation (5), where σ is the standard deviation of the blank sample measurement. S is the slope of the calibration plot. In addition, the red line in Figure 7 shows the calibration curve and the slope can be obtained as 112.6. The limit of detection of the microfluidic impedance sensor was calculated as 0.0032 μg/mL and the corresponding impedance value was 306.68 kΩ.

### 3.4. Specificity Test

In this paper, we use Southern Bean Mosaic Virus (SBMV) Virus (SBMV), Arabis Mosaic Virus (ArMV), and Tobacco Ringspot Virus (TRSV) as non-target viruses to verify the specificity of the ToRSV test. Considering that the biosensor is a disposable consumable, three replicate experiments were performed using different microfluidic chips for the same concentration of 10 μg/mL of SBMV solution, ArMV solution, TRSV solution, and a mixture of four viruses of SBMV, ArMV, TRSV, and ToRSV, respectively. The average impedance values were calculated for the three groups of experimental impedance values, and the standard error of the average impedance values were described by the error bars.

**Figure 8:**
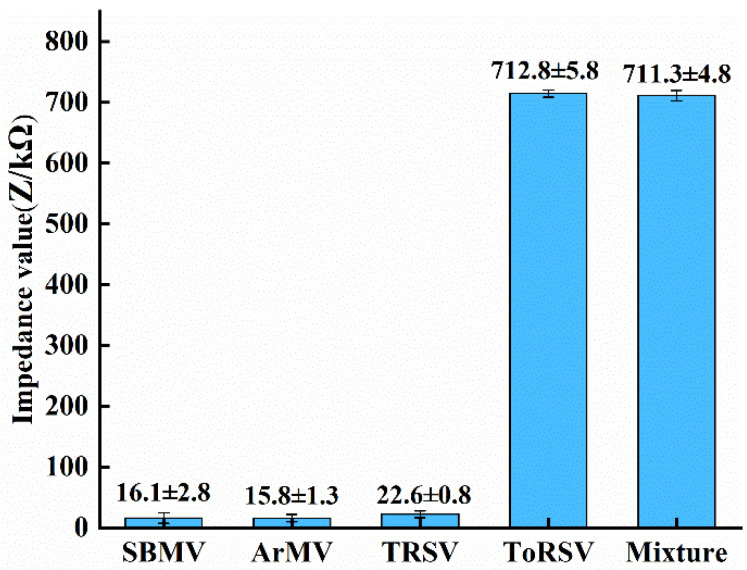
Impedance values of different viruses.

Figure 8 displays the impedance differences of the various viruses, including the impedance differences of the mixed four viruses. The impedance difference between the ToRSV solution and the mixture of four viruses is comparable. The impedance values of SBMV solution, ArMV solution and TRSV solution with the same concentration were close to those of the control group, which were greatly different from those of ToRSV solution. The experimental results show that the microfluidic impedance sensor designed in this paper for ToRSV detection has antibodies on the surface of the microfork finger electrode that react specifically only with ToRSV, and can recognize ToRSV from ToRSV single solution and various mixed solutions containing ToRSV, while it is ineffective in detecting other viruses. Therefore, it is demonstrated that the sensor has good specificity and effectiveness for ToRSV detection.

## 4. Conclusions

1. A microfluidic impedance sensor for ToRSV detection was designed. The ToRSV antibody was attached to the gold interdigital array microelectrode in the flow channel of the microfluidic chip. The combination of the antibody and ToRSV caused the impedance to change, and electrochemical impedance spectroscopy was made.
2. For the study of the mechanism of ToRSV impedance creation and variation, a new equivalent circuit model is suggested. ToRSV at concentrations of 0.001~1 μg/mL was used for impedance detection, and it was shown that the impedance spectrum obtained from the microfluidic impedance sensor and the impedance spectrum produced by fitting the equivalent circuit model were in good agreement.
3. The ideal detection frequency was determined using the impedance difference, and the results showed that the ideal detection frequency for this impedance biosensor to detect ToRSV concentration is 10.7 Hz. The ToRSV concentration and impedance value calculation model were built, and the detection specificity was evaluated. The experimental results reveal that the developed microfluidic impedance sensor has a detection limit of 0.0034 μg/mL, and good specificity and validity.
